# The Mechanical Behavior of *HAVAR* Foils Using the Small Punch Technique

**DOI:** 10.3390/ma10050491

**Published:** 2017-05-03

**Authors:** Shlomo Haroush, Daniel Moreno, Ido Silverman, Asher Turgeman, Roni Shneck, Yaniv Gelbstein

**Affiliations:** 1 Nuclear Research Center-Negev, P.O. Box 9001, Beer-Sheva 84190, Israel; ashertu10@walla.co.il; 2 Department of Materials Engineering, Ben-Gurion University of the Negev, Beer-Sheva 84105, Israel; roni@bgumail.bgu.ac.il (R.S.); yanivge@bgu.ac.il (Y.G.); 3 Soreq Nuclear Research Center, Yavne 70600, Israel; dmoreno@netvision.net.il (D.M.); ido.silverman@gmail.com (I.S.)

**Keywords:** small punch test, SPT, ball punch test, BPT, mechanical behavior, Co-based alloy, *HAVAR*, TEM

## Abstract

Prediction of the mechanical behavior of thin foils (~25 µm) requires special characterization techniques. The current work is focused on the mechanical and microstructural characterization of 25 µm *HAVAR* alloy foils following annealing, cold rolling, and subsequent heat treatments, using small punch testing (SPT), X-ray diffraction (XRD), and transmission-scanning electron microscopy (TEM). The SPT technique revealed that the annealed specimens exhibited the largest maximal load to failure and deformation (more than two-fold), compared to the cold rolled and heat treated conditions. The microscopy observations revealed high dislocation density following cold rolling and subsequent heat treatments. Following annealing, a cubic crystallographic structure (FCC) with equiaxed grains and a limited dislocation population was observed. Following cold rolling and subsequent thermal treatment, a preferred orientation texture (i.e., ‘deformation texture’) was observed with a very high dislocation density. The correlation between the mechanical behavior and the microstructural observations is discussed in detail.

## 1. Introduction

The Small Punch Test (SPT), Ball Punch Test (BPT), Disk Bend Test (DBT) [1], and Shear Punch Test [2,3,4,5,6,7] are common mechanical techniques for the characterization of small dimension or thin specimens. These testing methods are usually conducted on specimens that are too small to undergo standard tension tests. The SPT concept is based on locking a thin sheet-like specimen between two dies and pushing a punch against it with a spherical cap, up until failure. During the test, the load and the punch stroke are monitored simultaneously until the end-test criterion (e.g., maximal or failure load) is achieved. Many of the common standards, including the E-643 ASTM standard [8], are valid for specimens whose thickness ranges from 200 to 2000 μm, resulting in many research publications focusing on mechanical characterization of alloys thicker than 200 μm [9,10,11,12,13].

**Figure 1:**
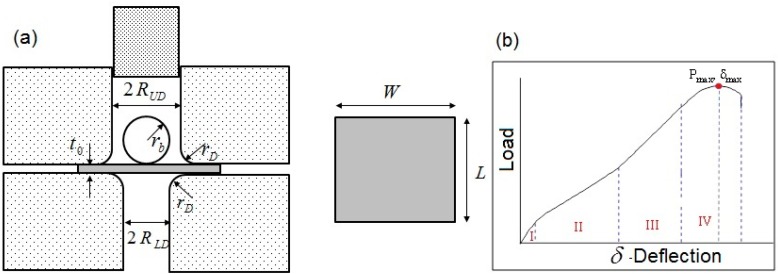
(**a**) Schematic of apparatus for small punch test of specimen of dimensions 8 × 8 mm, t_0_ = 25 µm. Adapted from [14], with permission from © 2015 Elsevier; (**b**) Schematic load vs. deflection curve for specimens thicker than 300 µm.

A schematic of apparatus for SPT and a typical load displacement curve are shown in Figure 1. The load displacement curve shown in Figure 1b is composed of four distinct regions, typical to highly ductile materials, as described in [10,11,12]: I—Elastic behavior, II—Plastic behavior (strain hardening), III—Plastic membrane stretching, and IV—plastic instability. In the vicinity of the maximal load, cracks are expected to develop in the specimen, followed by ductile propagation and fracture. The zone between regions I and II is used to estimate the material yield stress while the ultimate stress and fracture strain are estimated from zone IV. Many of the reported studies have focused on specimens having thickness values of 500 μm. Haroush et al. [14] have shown that for specimens having a thickness greater than 300 μm, classical plate equations can be used to estimate the yield stress, while for thinner specimens yield stress estimation from SPT is a very complex issue, since it is a function of the specimen thickness.

Thin foils having thickness values of 100 µm and below are used in a variety of applications such as for food service, shielding, vacuum chambers (Al), for electronics, batteries, circuit board, cable wraps (Cu), for aerospace, surgical instruments, tool wraps, heat exchangers (stainless steel), and more. In medical applications, a 25 µm thin foil is required as a window for fluorodeoxyglucose (FDG) production [15,16].

*HAVAR* is a high strength, non-magnetic, and corrosion resistant cobalt base alloy, suitable for operations at temperatures of up to 500 °C [17]. The alloy–composed of Co (42%), Cr (19.5%), Fe (19.1%), Ni (12.7%), Mo (2.2%), W (2.7%), and C (0.2%) atoms–is known to exhibit a cubic crystallographic structure (FCC) with a lattice parameter of 3.582 Å. One of its main applications is in the production of the short lived, positron emitting ^18^F isotope, typically produced by the reaction ^18^O(p, n) ^18^F. With the increasing demand for ^18^F by hospitals, an accelerated production rate is required. Thin (25 μm) foil of *HAVAR* is used in cyclotron accelerators as a window for liquid and gas targets. Irradiation of targets by proton or deuteron beams is carried out at various amounts of energy and occasionally at high currents and for long time durations, up to a few hours. During such irradiation conditions, the *HAVAR* foil might be damaged; its microstructure and mechanical properties can be degraded and, therefore, the window life-time can be significantly reduced.

The changes in *HAVAR* mechanical properties due to proton irradiation requires the characterization of its properties before and after the irradiation process in order to analyze the alloy’s ability to withstand the process. In particular, vendor instructions are to replace the foils every 1 mA-h, which can be translated to about a month of irradiation in the cyclotron.

The goals of the current stage of the research were to study the *HAVAR* foil microstructural and mechanical behavior in three metallurgical conditions; annealed (Ann.), cold rolled for 85–90% area reduction (CR), and cold rolled following subsequent heat treated at 500 °C for 3.5 h (CR & HT). The specimens were characterized by X-ray diffraction (XRD), microscopy observations, and SPT. The damage to the *HAVAR* foil as a function of proton energy and as a function of the integrated current will be studied at a later stage. We note that since the *HAVAR* foil during irradiation is exposed to a Gaussian shaped intensity beam, the damage and the temperature profile is non-uniform throughout the *HAVAR* window. Therefore, at locations where the temperature is higher, the increased thermal atom diffusion (annealing) may reduce the damage.

## 2. Experimental Procedure

**Figure 2:**
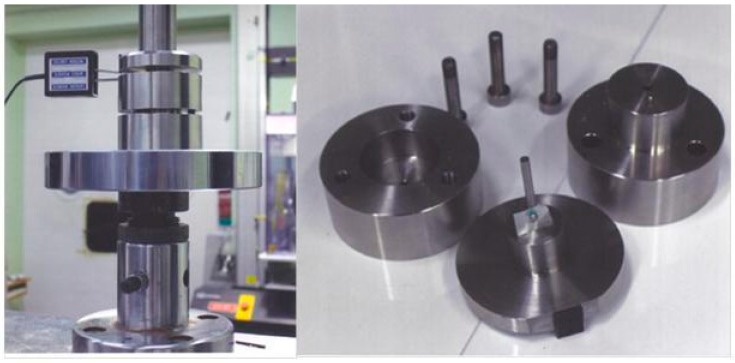
SPT apparatus used in this study.

25 µm-thick *HAVAR* foils (purchased from Hamilton Precision Metals, Lancaster, PA, USA) in the annealed form, cold rolled for 85–90% area reduction and subsequently thermally treated at 500 °C for 3.5 h were cut by scissors to an area of 8 × 8 mm, polished to 1µm surface finish, and cleaned by alcohol. Small punch testing was conducted using the apparatus shown in Figure 2 by the following steps: (1) clamping of the specimen in between the dies under 300 N; (2) pre-loading up to 5 N and balancing the stoke transducer (Instron COD—Crack Opening Displacement); (3) pushing the punch into the specimen under stroke control at a speed of 0.2 mm/min up to failure, as was indicated by the load drop. The punch used for the experiments was ball-shaped with a diameter of 2.4 mm, while the bottom die diameter was 3.0 mm. The ball adhered to the punch due to a thin layer of grease that was smeared on it, in order to center it in relation to the die and the test specimen. After failure, fractured surface was characterized by a Scanning Electron Microscope (SEM, JEOL JSM 5600, JEOL, Welwyn Garden, UK). Following each of the examined metallurgical conditions, the crystallographic structure was identified by XRD using a 1.5406 Å wavelength and a Cu tube.

The microstructure was investigated by using a JEOL 200 KV TEM (JEOL). For this purpose, the specimens were prepared by: cutting of 3 mm disks and selective etching using Struers Tenopol 5 with 10% Perchloric Acid + 90% Methanol.

## 3. Results and Discussion

### 3.1. Small Punch Test

**Figure 3:**
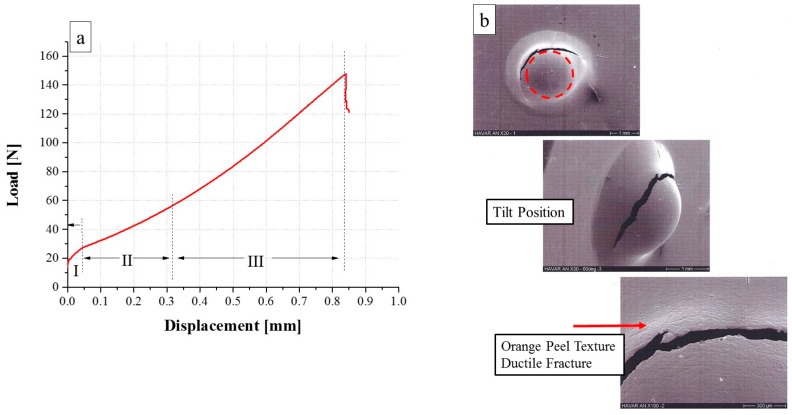
(**a**) Load versus displacement curve of 25 µm annealed *HAVAR* foils; (**b**) SEM fractography.

**Figure 4:**
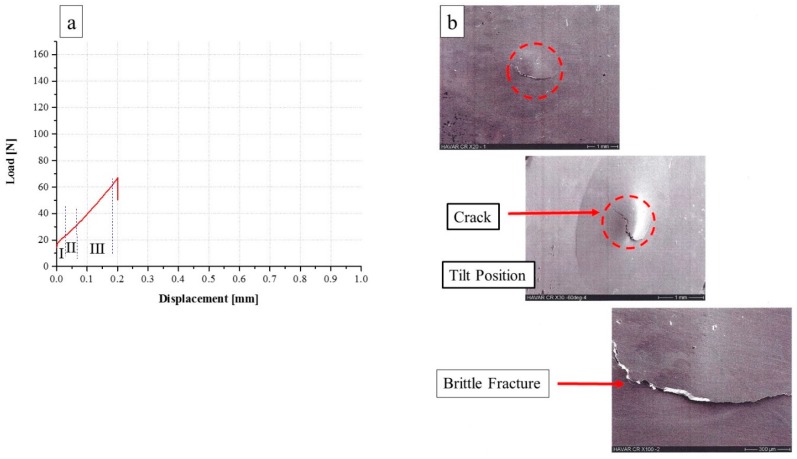
(**a**) Load versus displacement curve of 25 µm CR *HAVAR* foils; (**b**) SEM fractography.

**Figure 5:**
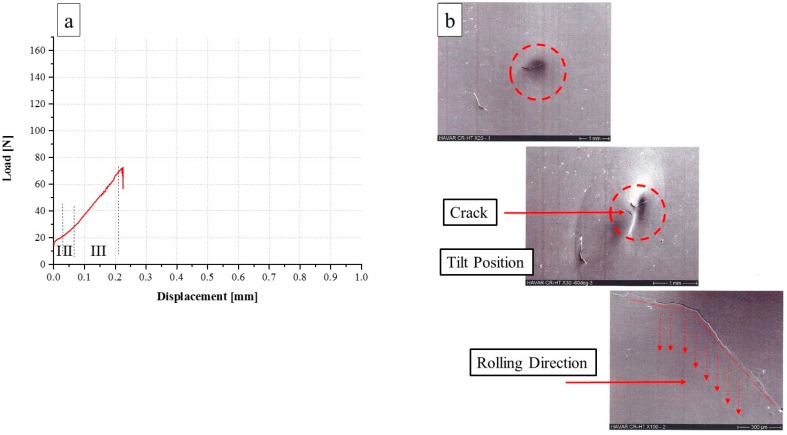
(**a**) Load versus displacement curve of 25 µm CR-HT *HAVAR* foils; (**b**) SEM fractography.

The SPT load and displacement curves and the fracture modes following the Ann., CR, and CR-HT conditions are shown in Figure 3, Figure 4 and Figure 5, respectively. It can be seen that for the Ann. condition (Figure 3a), three regions were apparent: I—elastic, II—strain hardening, and III—membrane stretching, were apparent, the fourth region described in relation to Figure 1b was absent, which indicates plastic instability. After stretching, the load dropped sharply, indicating a failure. The maximal load (*P*_max_, Figure 1b), was originally considered as the end-test criterion for all of the specimens. However, due to the different observed mechanical failure regimes following each of the metallurgical conditions tested, the displacement at maximum load (δ_max_, Figure 1a) was chosen as the characteristic parameter for comparison. The SPT results for all of the specimens are summarized in Table 1.

The fracture mode of the Ann. specimens is shown in Figure 3b. It can be seen than the fractured zone is surrounded by an ‘orange peel’ texture, indicating ductile behavior. This behavior is supported by the very high displacement and load values in the SPT curve and the final specimen’s shape shown in the figure.

From the SPT behavior of the CR condition (Figure 4a), a totally different behavior compared to the Ann. condition (Figure 3a) can be seen. Following CR, similarly to the Ann. condition, the same three mechanical regimes are observed, the maximal load displacement is smaller by about a factor of 4: 0.19 mm compared to 0.87 mm. Furthermore, the maximal load is smaller by about a factor of 2 (65 N), compared to 154 N. The end of test behavior is similar to in the Ann. condition—sharp load drop and failure. The fracture mode is shown in Figure 4b. It can be seen that the fractured zone is devoid of any ‘orange peel’ texture; it is focused on the specimen center and contains a crack propagated from the center to the edges while crossing the rolling direction. The fracture contour exhibits a very sharp edge without any evidence of plasticity. This conjecture is supported by the specimen shape at the end of the test, as can be seen in the figure. The displacement at maximum load and the maximum load values are summarized in Table 1.

From the SPT behavior of the CR-HT condition, shown in Figure 5a, a similar behavior to the CR condition (Figure 4a) and totally different behavior from the Ann. condition (Figure 3a) can be seen. In this case, the maximal load displacement and the maximum load values are very similar to for the CR specimen and smaller than for the Ann. The fracture zone, shown in Figure 5b, is located at the center of the sample and contains a crack propagated from the center to the edges without any evidence of plasticity, in a similar way as with the CR condition described above. It can be noticed that the crack propagates in a random orientation, originally with an angle to the rolling foil direction that changed almost perpendicular towards the direction of the rolling (Figure 5b).

The tensile mechanical properties of the Ann., CR, and CR-HT conditions, reported by the manufacturer, are summarized in Table 2 [17]. It can be seen that the alloy in the Ann. condition is very ductile (e~40%), with yield and ultimate stresses of about 500 (S_0.2%_) and 1000 (S_UTS_) MPa respectively. For the CR condition, the yield and ultimate stresses almost doubled, but the elongation to fracture is very limited. For the CR & HT condition, the reported yield and ultimate stresses are 25% higher than the CR and more than doubled compared to the Ann. conditions, while the elongation to fracture is also very limited.

According to the previously reported tensile mechanical properties (Table 2) and the currently examined SPT behavior (Table 1), it can be seen that the tensile elongation to fracture and the SPT maximal load displacement (δ_max_), can be correlated. The Ann. specimen exhibits the largest tensile elongation to fracture (e) and the highest maximal load displacement. On the other hand, the CR and CR-HT specimens had very limited elongation to fracture and the displacement in the SPT was smaller by about a factor of 4 compared to the Ann. The displacement of the CR and CR-HT can be considered the same.

### 3.2. Crystallographic Structure

**Figure 6:**
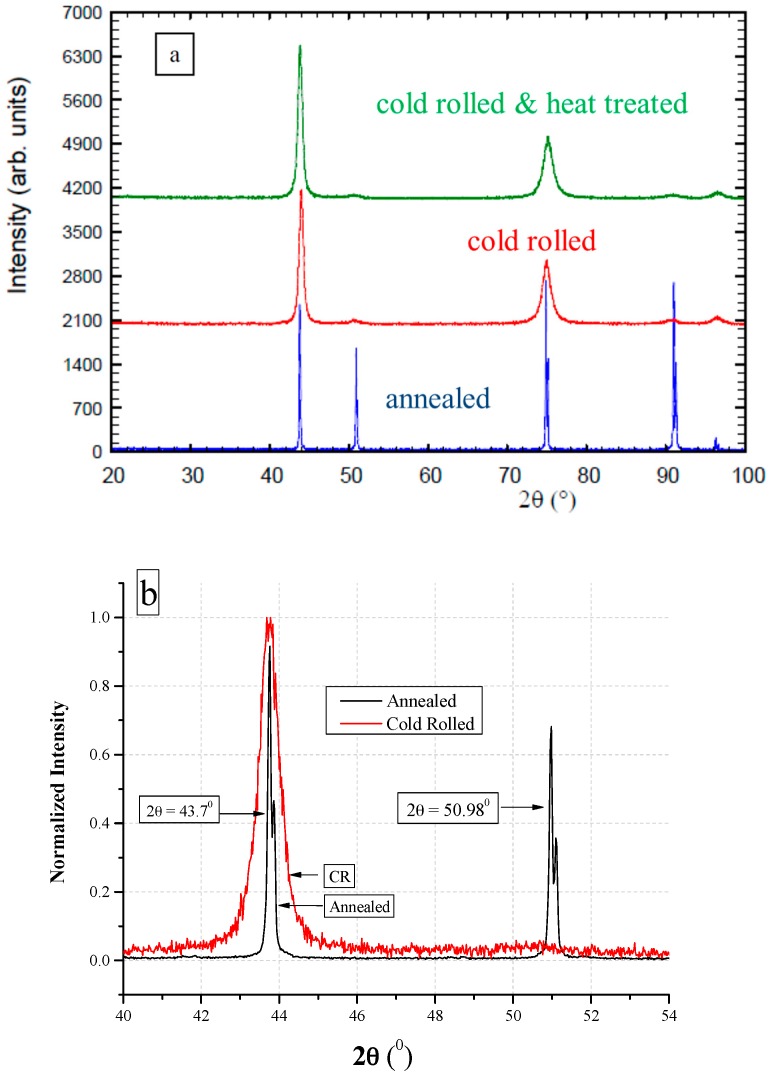
XRD diffractogram of *HAVAR* (**a**) following the three investigated metallurgical conditions; (**b**) following Ann. and CR at 2θ = 43.7° and 51°.

The XRD results following all of the tested conditions are shown in Figure 6. It can be seen that the Ann. condition exhibits sharp and narrow peaks, indicating specimens free of internal stresses, while the CR-HT specimens exhibit much broader peaks, some with low intensity and some are missing (2θ = 51°, 91.5°), indicating high internal stresses and preferred orientation. The crystallographic structure of *HAVAR* for all of the investigated metallurgical conditions was found to be FCC with a lattice parameter of 3.58 Å. Figure 6b highlights the differences between the Ann. and CR peaks at 2θ = 43° and 51°.

### 3.3. TEM Observation

**Figure 7:**
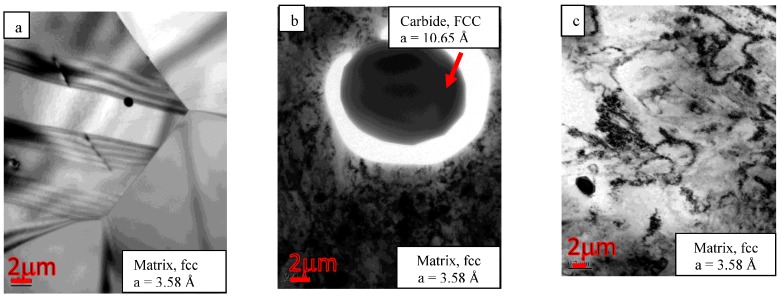
TEM observations of *HAVAR* in three metallurgical condition: (**a**) Annealed, microstructure of three grains, FCC, 3.58 Å; (**b**) CR, M_23_C_6_ carbide, FCC, 10.65 Å; (**c**) CR-HT.

**Figure 8:**
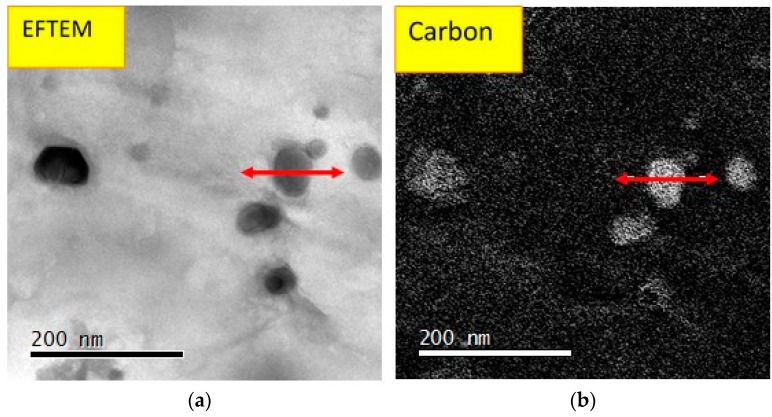
EFTEM image of CR-HT at 800 °C for 1 h, carbon element: (**a**) bright field image of the matrix and carbide phases; (**b**) carbon profile over the carbide.

The TEM studies have shown that the microstructure of the annealed foil contains equiaxed grains about 4 µm in diameter, a low population of dislocations in the bulk and a small quantity of scattered carbides (Figure 7a). Electron diffraction confirmed the XRD results and showed that the matrix is cubic structured (FCC) with a lattice parameter of 3.58 Å. The TEM studies revealed that all of the observed carbides across the thickness are enriched (53–69 wt %) by Cr in comparison to 20 wt % Cr in the matrix as shown in Figure 8. Furthermore, the observed carbides were found to contain most of the *HAVAR* alloying elements. The element concentrations in the matrix and carbide phases are summarized in Table 3. Electron diffraction and energy filtered mapping (EFTEM) of the carbide phase (Figure 8) revealed a cubic crystallographic structure (FCC) with a lattice parameter of 10.65 Å with much higher carbon content compared to the matrix. The best fitted carbide was found to be a M_23_C_6_ type, such as (Cr,Co,Mo,Ni,W)_23_C_6_ or Fe_21_W_2_C_12_ with a lattice parameter of 10.622 Å. These observations are in contrast to Benson’s report [18], which concluded that heat treatment of *HAVAR* following cold working results in hexagonal WC precipitates (epsilon phase).

Following CR, a preferred orientation (‘deformation texture’) (Figure 6b) was observed, in agreement with the XRD analysis, with a very high dislocation density compared to the Ann. condition (Figure 7b). Electron diffraction revealed that the crystallographic structures of the matrix and carbide phases are similar to those of the Ann. condition.

Bright field TEM image following the CR-HT condition is shown in Figure 7c. The presence of carbides in addition to the high dislocation density can be easily seen. According to Hamilton’s data sheet [17], the tensile properties of the CR-HT are higher than the CR by about 25%.

## 4. Conclusions

The mechanical properties of 25 µm thick *HAVAR* foils following annealing, cold rolling, and subsequent 500 °C for 3.5 h thermal treatment were characterized by the SPT and correlated to the microstructural characteristics. According to the overall observations, we conclude that *HAVAR* can be strengthened in two stages: cold work and heat treatment following cold working. Cold working is the primary strengthening mechanism while the heat treatment is the secondary mechanism. Cold working created a variety of defects account for the strengthening mechanisms, namely: very high dislocation density, stacking faults, twins, sub-twins, and the coexistence of two crystallographic phases (Matrix, (γ) FCC and (ε) HCP phase), evolved during the cold rolling into a mosaic-like texture. Heat treatment following cold working promoted the precipitation of fine cubic M_23_C_6_ carbides in the range of a few nm in diameter as a secondary strengthening mechanism. These carbides precipitate during the heat treatment and contribute to the alloy strengthening. In all of the investigated conditions, a *HAVAR* matrix was observed; exhibiting an FCC crystallographic structure with a lattice parameter of 3.58 Å, with embedded FCC-M_23_C_6_ type carbides and a lattice parameter of 10.65 Å, containing almost all of the matrix elements.

The changes in microstructure due to cold rolling and heat treatment can explain the huge changes in the mechanical properties. The annealed state has an equi-axed grain size of about 2–5 µm diameter, containing a few dislocations and a few twins. This microstructure exhibited the highest load capacity and ductility among all of the investigated metallurgical conditions. On the other hand, cold rolling with and without subsequent thermal treatment has a very high density of defects, limiting the dislocation mean path, while increasing the yield stress and reducing the ductility toward a brittle failure morphology.

## Figures and Tables

**Table 1:** Maximal load displacement and maximum load of 25 µm Ann., CR, and CR-HT *HAVAR* foils.

Maximal Load Displacement (mm)	Load (Max.) (N)	Alloy
0.87 ± 0.01	154.19 ± 0.8	Ann.
0.19 ± 0.01	64.83 ± 3.4	CR
0.22 ± 0.007	69.62 ± 3.3	CR-HT

**Table 2:** Typical tensile mechanical properties of *HAVAR*, as were reported by the manufacturer [17].

Typical Tensile Mechanical Properties of HAVAR			
	Ann.	CR	CR-HT
S_UTS_ (Mpa)	960	1860	2275
S_0.2%_ (Mpa)	480	1725	2070
Elongation to Fracture (%)	40	1%	1%
Hardness (HRc)	25	50	60
Modulus of Elasticity (Gpa)	200	200	200

**Table 3:** Element concentrations in the matrix and carbide phases.

Element (wt %)	Co	Co	Cr	Ni	Fe	W	Mo	Mn	C
Matrix	41.68	41.68	19.49	12.5	19.5	2.78	2.19	1.56	0.20
Particle (carbide)	12–14	12–14	53–69	3–4	7–10	5–6	7–10	1.5–2.5	Higher than 0.2
